# Chloroform—the New Anæsthethic Agent

**Published:** 1848-02

**Authors:** 


ARTICLE III.

### CHLOROFORM—THE NEW ANÆSTHETIC AGENT.

Our exchange journals, received since our last issue, are full of reports from various sources, and correspondence between eminent medical men, on the subject of the anæsthetic agent—chloroform.

In our last number, we laid before our readers all that had been received by us on the subject, which, however, only amounted to an announcement of the discovery and a very faint outline of its effects. The amount of matter now before us, which has accumulated since then, is so great, that were we to give but a part only of them in our department of selected matter, we should have but little room for other matter. We shall accordingly endeavor to present, in a condensed form, the most important results that have been deduced by observation and experiments, in regard to the history of chloroform; its uses in the different departments of practice; effects compared with ether; the best mode of inhalation ; its physiological action, and the dangers to be apprehended from its use, &c., &c.

*Composition.*—Chloroform is chemically the "Sesquichloride of Formyle." Formyle is the hypothetical radical of formic acid, which takes its name from having been first obtained from the red ant. Formic acid contains carbon 2 equivs., hydrogen 1, oxygen 3. Replace the three equivalents of oxygen by chlorine, and we obtain C 2, H CL 3, the composition of chloroform.

*History.*—It was discovered in 1831, by E. Soubeiran, chief pharmacian to the hospitals of Paris ; about the same time by Liebig, in Germany, and Mr. Guthrie of our own country. A number of the foreign reports fail to mention the name of Mr. Guthrie as one of the discoverers of chloroform. An account of his discovery was published in Silliman's Journal, (in vols. 21, 22, 1832,) and is mentioned in Wood & Bache's Dispensatory, under the head of "chlorine ethers," in the appendix to the edition published in 1839.

While others are contending for the honors of the discovery, American journalists should not allow the merits of their own countrymen to be overlooked. The chemical constitution of chloroform was first investigated by Dumas, who also assigned to it its present name. Flourens is said, by the French, to have been the first to describe its anæsthetic properties. In a paper presented to the Academy, March 8, 1847, he announces the effects of the inhalation of chloroform on a rabbit. He says at the end of some minutes it "was entirely etherized." "The spinal marrow was exposed and the posterior cords were insensible." Three out of the five of the anterior cords "had lost their *motricité*. Professor Ives and Dr. N. B. Ives, of New Haven, speak favorably of its effects internally. The U. S. Dispensatory *(loc cit.)* has this remark : "In affections characterized by difficulty of respiration it may be used by *inhalation.*" All agree, however, in awarding to Prof. Simpson, of Edinburg, the merit of having been the first to use it on man for producing insensibility to pain ; to him alone, then, is due its general introduction as an anæsthetic agent. Dr. S. having used the ether in obstetrical practice, found it liable to several objections; a liability to produce bronchial irritation; its disagreeable and persistent odor, &c., &c. He accordingly tried a number of other volatile fluids said to possess agreeable odors, among others, at the suggestion it is said of Mr. Waldie, chloroform, which alone gave perfect satisfaction. The first public notice of the new agent was made by Prof. S. in a paper read before the Medico Chirurgical Society of Edinburg, on Nov. 10, 1847.

*Preparation and mode of determining its purity.*—Chloroform has been prepared by several different processes. The most economical and simplest, is by the action of chloride of lime upon alcohol, pyroxylic spirit, or acetone. The essentials to its production appear to be the action of chlorine on certain hydro-carbonsin the presence of an alkaline base. It is highly important that the product should be carefully freed from the presence of all other compounds. Professor Simpson's formula is perhaps the best. We have used it in our own experiments and find it succeeds well. We give it as communicated by Prof. Simpson to Prof. Meigs, of Philadelphia.

"The following is the formula for chloroform, communicated by Professor Simpson:

| | | |
|---|---|---|
| "Take of Chloride of Lime in powder, | | 4 pounds. |
| Water, | - - - | 12 " |
| Rectified Spirit, | - - | 12 fluid ounces |
| | | "Dumas." |

The cloride of lime and water being first well mixed together, the spirit is added. Heat is then applied to the still, (which ought not to be more than a third full,) but as soon as the upper part of the still becomes warm, the heat is withdrawn and the action allowed to go on of itself. In a short time the distillation commences, and whenever it begins to go on slowly the heat is again applied. The fluid which passes over separates into layers, the lower of which is chloroform. This, after having been separated from the weak spirit forming the upper layer, is purified by being mixed with half its measure of strong sulphuric acid, added gradually. The mixture, when cool, is poured into a leaden retort, and distilled from as much carbonate of baryta by weight, as there is sulphuric acid by measure. The product should be allowed to stand over quicklime for a day or two, and repeatedly shaken and then redistilled from the lime."

In conducting the above process a very capacious vessel must be used ; the materials foaming very much during the escape of the chloroform. The vessels used should be of glass, earthenware, wood or lead, for the first process, and of glass for the purification. In the specimens we have ourselves prepared and used, we have omitted the distillation with sulphuric acid. The only impurity we have to suspect being alcohol, the presence of which we are well satisfied is the principal cause of the unpleasant, dangerous and stimulating effects of the impure specimens, we have uniformly adopted washing with distilled water with subsequent distillation, at a very gentle heat, as the sole mode of purification, and have reason to believe that a good article is thus obtained. The washed chloroform may be separated from the water, by subsidence and withdrawal by a pipette, the lower stratum being of course the chloroform. It is stated in a French paper that the stinging sensation which the chloroform produces upon the lips and other delicate parts with which it may omce in contact, results from the presence of absolute alcohol. The same report also states that the best test for the purity of the article is derived by dropping the chloroform into water and agitating it. If pure it subsides to the bottom, remaining transparent, and leaving the water, also, transparent; if impure a milky cloud is produced in one or both strata. We recommend our readers to apply this test before using any specimen they may obtain, as the high price of the pure article may render it an object to adulterate it.

*Comparison of the effects of Chloroform and Ether.*—Prof. Simpson, in a communication to the Medico-Chirurgical Society, of Edinburg, thus compared the effects of these two agents.

"As an inhaled anæsthetic agent, it possesses, I believe, all the advantages of sulphuric ether, without its principal disadvantages.

1. A greatly less quantity of chloroform than of ether is requisite to produce the anæasthetic effect, usually from a hundred to a hundred and twenty drops of chloroform being sufficient, and with some patients much less. I have seen a strong person rendered completely insensible by seven inspirations of thirty drops only of the liquid.

2. Its action is much more rapid and complete, and generally more persistent. I have almost always seen from ten to twenty inspirations suffice, sometimes fewer. Hence the time of the surgeon is saved ; and that preliminary state of excitement which pertains to all narcotizing agents being curtailed, or, indeed, practically abolished, the patient has not the same degree of tendency to exhilaration and talking.

3. Most of those who know from previous experience the sensations produced by ether inhalation, and who have subsequently breathed the cloroform, have strongly declared the inhalation and influence of chloroform to be far more agreeable and pleasant than those of ether.

4. I believe that, considering the small quantity requisite as compared with ether, the use of chloroform will be less expensive than that of ether, more especially as there is every prospect that the means of forming it may be simplified and cheapened.

5. Its perfume is not unpleasant, but the reverse ; and the odor of it does not remain for any length of time attached to the clothes of the attendant, or exhaling in a disagreeable form from the lungs of the patient, as so generally happens with sulphuric ether.

6. Being required in much less quantity, it is much more portable and transmissible than sulphuric ether.

7. No special kind of inhaler or instrument is necessary for its exhibition. A little of the liquid diffused upon the interior of a hollow-shaped sponge, or a pocket-handkerchief, or a piece of linen or paper, and held over the mouth and nostrils so as to be fully inhaled, generally suffices in about a minute or two to produce the desired effect."

All observers, whom there is reason to believe have used a *pure* article, coincide with these remarks of Prof. S. ; where a difference is observable in the reports, it in all probability is referable to the use of an adulterated article or to a difference in the mode of inhalation. The mode of inhalation prefered by the discoverer, it will be perceived, avoids the use of all instruments. He prefers as stated by him in other places the silk pocket handkerchief made up into a cup-shape. The use of the inhaler we can readily conceive may produce bad effects in two ways:

1. By not admitting sufficiently freely the oxygen of the air, and, consequently, producing partial asphyxia, together with the normal effects of the chloroform.

2. By permitting the accumulation in the sponge of the instrument, of adulterations less volatile than the chloroform.

In the latter case, the first individuals would inhale the almost pure vapor and experience effects more or less nearly approaching to normal, accordingly as the specimen was more or less pure. Those, however, who should inhale from the instruments after prolonged use, would breathe a mixed vapor of chloroform and of the less volatile liquids present, from which the pure effect could scarcely be expected. We have frequently breathed by means of a tube, from a bottle containing a solution of chloroform in alcohol; the first inspirations had the odor, taste and effects of almost pure chloroform, but after a time, the vapor of alcohol predominated and ultimately was left by itself.

*Uses of Chloroform in the different departments of practice of Medicine and Surgery.*—The advantages derived from the use of the agent in *surgical operations* is now established beyond controversy. The journals are full of evidence on this subject, from the hospitals on the continent, in the British Isles, and in America. It is stated that aside from the freedom from pain, patients recover more rapidly, and the average success of operations is increased.

In *obstetrical practice* the evidence is strong in favor of its use. Professor Simpson states that recently he has used it, with few and rare exceptions, in every case of labor which has been under his care, and the results have been most happy and gratifying. "I never," says he, "had the pleasure of watching over a series of more perfect or rapid recoveries; nor have I once witnessed any disagreeable results to either mother or child." Reports from other sources bear testimony to the same.

In *Insanity*, though evidence is wanting to prove chloroform a *curative* agent in chronic cases of mania, it is shown to be exceedingly valuable. In cases of dementia with excitement and wakefulness it has produced a good effect.

In *Delirium Tremens* it has produced critical sleep and apparent cure. In *puerperal mania* it has procured sleep and calmed the excitement so as at least to aid in the cure.

Professor Simpson recommends it as an *antispasmodic* in asthma, laryngismus, tetanus, hooping-cough, dysmenorrhœa, colic, and the pain attending the passage of biliary calculi. As a *diffusible stimulant*, in *small doses*, to arrest the commencement of ague, in hysteria, &c., and as a narcotic in neuralgia.

*Physiological action.*—A committee of the Edinburg Medico-Chirurgical Society, after an extensive series of experiments on men and animals, report, that chloroform inhalation like that of ether, seems to produce loss: "1. Of the cerebal functions; 2. Of the spinal functions; and, thirdly, of those of the medulla oblongata." The loss of consciousness is referred to the effect on the cerebrum, and occurs first in point of time; if the inhalation continues, spinal symptoms appear, such as tetanic spasms, involuntary evacuations, and hysterical symptoms; and, if pursued too far, respiration and the heart's action may become so enfeebled as to result in death. In all the animals killed by it, the right side of the heart was engorged with venous blood. The effects upon the blood require yet to be examined more fully, nothing determinate having yet been announced.

*Danger attending its use.*—From the effect on animals there remains no doubt that chloroform may produce death. It is consequently a curious fact that so few instances of bad effects have resulted from its indiscriminate use, even for purposes of amusement and by uninformed persons. Though several newspaper paragraphs have met our eye mentioning unpleasant, and even fatal effects, we have before us, in professional journals, but a single fatal case,—that of Mrs. Simmons, of Cincinnatti. We had intended giving the report in full, but our space will not admit it.

It may be found in the Western Lancet, of March, 1848. Death resulted within ten minutes from commencing to inhale, apparently, from the effect on the heart and respiratory muscles. The brain and lungs, though somewhat, were not extensively congested; general aspect, color, and consistence of the brain normal; pleura highly injected with some effusion; heart flaccid *and all its cavities entirely empty;* the *aorta, pulmonary artery and cava all eupty;* blood fluid as water in every part of the body; not a coagulum was seen in any vessel; globules altered; color in every part of the system was that of dark venous blood. The chloroform was of specific gravity, but 1. 3. contained some alcohol, and was applied with Morton's inhaler. We do not wish to pre-judge in the case, but we are inclined to refer the bad effects to the alcohol accumulated in the manner we have expressed above.

*Antidote to the effects.*—None has yet been mentioned. We have been informed by Mr. B. H. Bartlett, of this city, (Chicago,) that he has found morphiæ sulph. placed upon the tongue and allowed to dissolve in the mouth, to remove the effects, very rapidly, of both ether and chloroform. He uses ½ gr. repeated every few minutes. Before our next we will test the value of the suggestion.

We subjoin the remark, (omitted in its proper place,) that the chloroform should have a specific gravity of 1.48. M. Soubeiran, in a late memoir read to the Academy of Sciences, in Paris, "tells us that a rapid method of approximating to this, is by making equal parts of strong sulphuric acid and water, and allowing the mixture to cool. A few drops of the chloroform poured into the fluid ought to sink to the bottom, if sufficiently pure for medical use; but if they float on the surface, the chloroform should be rejected."

J. V. Z. B.

